# Machine Learning Prediction of Nitrification From Ammonia- and Nitrite-Oxidizer Community Structure

**DOI:** 10.3389/fmicb.2022.899565

**Published:** 2022-07-11

**Authors:** Conard Lee, Fatemeh Amini, Guiping Hu, Larry J. Halverson

**Affiliations:** ^1^Interdepartmental Microbiology Graduate Program, Iowa State University, Ames, IA, United States; ^2^Department of Plant Pathology and Microbiology, Iowa State University, Ames, IA, United States; ^3^Department of Industrial and Manufacturing Engineering, Iowa State University, Ames, IA, United States

**Keywords:** nitrifiers, machine learning, nitrification, microbiome, ammonia-oxidizers

## Abstract

Accurately modeling nitrification and understanding the role specific ammonia- or nitrite-oxidizing taxa play in it are of great interest and importance to microbial ecologists. In this study, we applied machine learning to 16S rRNA sequence and nitrification potential data from an experiment examining interactions between cropping systems and rhizosphere on microbial community assembly and nitrogen cycling processes. Given the high dimensionality of microbiome datasets, we only included nitrifers since only a few taxa are capable of ammonia and nitrite oxidation. We compared the performance of linear and nonlinear algorithms with and without qPCR measures of bacterial and archaea ammonia monooxygenase subunit A (*amoA*) gene abundance. Our feature selection process facilitated the identification of taxons that are most predictive of nitrification and to compare habitats. We found that *Nitrosomonas* and *Nitrospirae* were more frequently identified as important predictors of nitrification in conventional systems, whereas *Thaumarchaeota* were more important predictors in diversified systems. Our results suggest that model performance was not substantively improved by incorporating additional time-consuming and expensive qPCR data on *amoA* gene abundance. We also identified several clades of nitrifiers important for nitrification in different cropping systems, though we were unable to detect system- or rhizosphere-specific patterns in OTU-level biomarkers for nitrification. Finally, our results highlight the inherent risk of combining data from disparate habitats with the goal of increasing sample size to avoid overfitting models. This study represents a step toward developing machine learning approaches for microbiome research to identify nitrifier ecotypes that may be important for distinguishing ecotypes with defining roles in different habitats.

## Introduction

Microbial communities mediate a variety of biogeochemical cycles that are important to agricultural productivity. For example, nitrification, the conversion of ammonia to nitrite and eventually to nitrate by ammonia- and nitrite-oxidizing bacteria, is greatly influenced by agricultural practices designed to limit nitrate leaching and contamination of waterways, particularly in the Midwest corn belt. Given its importance, there has been great interest in modeling nitrification and the role of specific nitrifier ecotypes (i.e., an ecologically distinct lineage within a traditional species classification) (Booth et al., 2005; Ward et al., 2006; Koeppel et al., 2008; Zhang et al., 2010; Verhamme et al., 2011), particularly the ammonia-oxidizers since they mediate the rate-limiting step (Hart et al., 1994). A greater understanding of the potential role of distinct ecotypes in nitrification could provide insights into how ecosystems shape nitrifier evolution and, consequently, their nitrifying properties.

As the number of soil and plant microbiome datasets grows, there is an increasing desire to use microbiome data to diagnose disease (Sze et al., 2018, 2019; Topçuoglu et al., 2020), predict crop yields (Wang et al., 2016; Chang et al., 2017; Shahhosseini et al., 2020, 2021), or model biogeochemical/ecosystem processes (Shahhosseini et al., 2019; Thompson et al., 2019). Biological models of complex systems have long relied on simple input–output models refined over the course of exhaustive experimentation. Models utilizing statistical inference offer only limited predictive capabilities, in part because of the inherent noisiness and high dimensionality of biological data, such as with 16S rRNA gene amplicon data. Machine learning provides one possible solution to these challenges and is increasingly used for modeling complex biological data (Pasolli et al., 2016; Chang et al., 2017; Thompson et al., 2019). The primary benefits of machine learning over more traditional statistical methods are that machine learning models do not rely on strict assumptions about the sources of data and the data itself, and they are capable of taking all factors into account despite noise and variation in data collection methods (Camacho et al., 2018). Additionally, the express purpose of machine learning models in this capacity is to predict outcomes based on given data, whereas statistical models are generally capable of inferring relationships between given data but are not designed to predict future outcomes (El Naqa and Murphy, 2015; Camacho et al., 2018). Regression-based machine learning provides a means to elucidate relationships between distinct ecotypes/taxons within a habitat and the ecosystem processes they mediate.

In this report, we used machine learning to model nitrification to determine whether specific nitrifier ecotypes can be identified as potential biomarkers for unique habitats or management regimes. Given the high dimensionality of microbiome data, we only included nitrifiers since they are capable of aerobic ammonia and nitrite oxidation, and there likely exist unique ecotypes that define nitrification rates. In soil, nitrifier taxa include *Nitrobacter* (α-Proteobacteria), *Nitrosomonadaceae* (β-Proteobacteria), *Nitrospirota, Nitrospinota*, and *Thaumarcheota*. Rather than focusing on a binary classification scheme, we used the relative abundance of each nitrifier OTU to predict a continuous variable, nitrification potential. In nitrification potential, nitrate production rates are determined under optimal conditions over a short period of time when substrate (ammonium) is not limiting. We assumed that nitrifier relative abundance measured using 16S rRNA gene profiling reflects the community's ability to nitrify. Here, we used rRNA amplicon microbiome data from a study that examined interactions between the cropping system and the rhizosphere on nitrification (Bay et al., 2021). The rhizosphere reflects plant rhizodeposition influences on the soil microbial communities' metabolic activities within 5 mm of the root. This is likely crucial to nutrient cycling since the plant is capable of influencing nutrient dynamics by directly absorbing nitrogen or by altering microbial community activities (Henneron et al., 2020). With this dataset, we explored the use of linear and nonlinear machine learning to model nitrification to identify nitrifier ecotypes specific to the rhizosphere or a cropping system. We also explored the benefits of including other metadata and the potential risks of merging datasets from distinct habitats.

## Materials and Methods

### Site Description and Data Collection

The soil used for generating the 16S rRNA amplicon data (Bay et al., 2021) used in this study was collected from the Iowa State University Marsden Farm's (Boone County, IA) long-term cropping system experiment. The Marsden site experiment examines three cropping systems differing in rotation complexity and fertilizer inputs; detailed descriptions of management practices and site properties were previously described (Davis et al., 2012). We collected soil from a conventional system consisting of a 2-year rotation of maize and soybean that receives inorganic N fertilizer and a diversified system consisting of a 4-year rotation of maize, soybean, oats/alfalfa, and alfalfa that receives composted cattle manure as fertilizer and an occasional inorganic N side-dress (Davis et al., 2012; Tamburini et al., 2020). These soils were used to fill rhizotrons (boxes that readily disassemble to access roots) to grow two maize plants to the V4/V5 developmental stage, when plants are poised to rapidly take-up N to meet their nutritional needs. We predicted that there would be greater coupling between plant roots and microbes, resulting in lower nitrification rates in the rhizosphere of diversified systems. In this study (Bay et al., 2021), we used one set of rhizotrons for obtaining bulk soil and rhizosphere samples for extracting DNA for generating 16S rRNA gene amplicon profiles of the microbial community and for quantitative PCR measurements of ammonia-oxidizing bacteria (AOB) and archaea (AOA) ammonia monooxygenase subunit A (*amo*A) gene abundance. The second set of rhizotrons was used for collecting rhizosphere soil from rhizotrons planted with maize and bulk soil from rhizotrons that were unplanted. The rhizosphere and bulk soil were used for determining the potential rates of nitrification as determined previously (Herman et al., 2003). The ammonia and nitrate pool sizes were determined by the ISU Soil and Plant Analysis Laboratory. AOA/AOB abundance, nitrification potential and gross rates, and nitrate/ammonia pool size results have been published previously (Bay et al., 2021).

### 16S rRNA Gene Sequencing Data

Bulk soil and rhizosphere samples were used for 16S rRNA gene sequencing of the V4 region (515F/806R primer set) to measure the relative abundance of microbial populations. In this study, the rhizosphere includes fine soil adhering to the root and the rhizoplane (root surface-soil interface) since the entire plant root was immersed into a buffer, vortexed, and sonicated prior to centrifugation to obtain a rhizosphere sample as described previously (Bay et al., 2021). The sequence data used in our analyses were generated by Bay et al. (2021), which are available through NCBI short read archive (PRJNA686799). Following the removal of adapters, mitochondria, chimeras, and singletons, quality filtering and OTUs clustering taxonomy were assigned at the 97% level using SILVA (version 128). In this dataset, there were approximately 2,500 conventional bulk soil, 2,800 diversified bulk soil, 2,250 diversified rhizosphere, and 2,500 conventional rhizosphere OTUs (Bay et al., 2021). From this OTU dataset, we extracted known ammonia- and/or nitrite-oxidizing bacteria (*Thuamarchaeota, Nitrosomondaceae, Nitrobacter, Nitrospinota*, and *Nitrospirota*); the δ-*Proteobacteria Nitrospinae* are now considered a separate phylum, the *Nitrospinota* (Luecker et al., 2013). This resulted in 226 OTUs from which a subset was used in our analyses based on the OTUs feature selection criteria outlined below.

### Study Design

For this study, we had 15–16 measurements (nitrifier OTUs, nitrification potential rates, qPCR data, ammonia, and nitrate pool sizes) for each of the conventional bulk soil, conventional rhizosphere, diversified bulk soil, and diversified rhizosphere. Consequently, there were 30–31 measurements (nitrifier OTUs, nitrification potential rates, qPCR data, and ammonia and nitrate pool sizes) for conventional cropping systems, diversified cropping systems, bulk soil, and rhizosphere.

### Feature Selection

For this study, features for the machine learning modeling included nitrifier OTUs and ammonia and nitrate pool sizes, as well as qPCR measurements of AOB and AOA *amo*A gene abundance in a separate analysis. Given the large number of nitrifier OTUs relative to the sample size for each analysis, we developed a hierarchical OTU feature selection process to reduce the number of nitrifier OTUs to minimize the potential for overfitting the model. This process included retention of an OTU if detected in all the samples, meeting a minimum taxon-specific relative abundance threshold (~1/50th of the most abundant OTU in that taxon), and whether data suggested the OTUs were potentially metabolically active based on RNA-based profiling (ribosome abundance) in the same soil sample (Bay et al., 2021). We did not include OTUs that were highly correlated (Pearson's correlation *R*^2^ ≥ ±0.8, *p* ≤ 0.05) with each other unless the OTUs were identified as a module or connector hub in separate network analysis (unpublished data); co-correlated OTUs with the highest relative abundance were retained. The dataset, which comprises 42 features (OTUs and meta-data) in each comparison, was then subjected to principal component analysis (PCA) to further reduce the number of features by retaining only those that collectively explain 80% of the data's variance. We also standardized each OTU by centering them on their mean and scaling them by their standard deviation, before the PCA (Abdi and Williams, 2010). The relative abundance of each OTU *k* in the sample *p* has been standardized according to the following equation:


$$\begin{array}{l} relative\;abundance\;of\;OTU_{standardized}^{k,p}=\;\frac{{relative\;abundance\;of\;OTU_{original}}^{k,p}-mean\;\left(relative\;abundance\;of\;OTU^{k}\right)}{std\;\left(relative\;abundance\;of\;OTU^{k}\right)} \end{array}$$

where mean (relative abundance of OTU^*k*^) and std (relative abundance of OTU^*k*^) are the mean and standard deviation of the relative abundance of *k*th OTU over all samples, respectively.

### Model Training and Evaluation

Supervised machine learning was used to predict nitrification potential from OTUs of known ammonia- and nitrite-oxidizers and ammonia and nitrate pool sizes with or without ammonia-oxidizer *amo*A gene abundance. Models were developed primarily using the *sklearn* package in Python (version 3.7 or later). We compared linear (multiple regression and support vector machine (SVM)) and nonlinear (decision tree and random forest) models. The k-fold cross-validation is used for both hyperparameters tuning and the performance evaluation of each prediction model. In k-fold cross-validation, data is split into k, equal-sized folds, and in each iteration, k-1 folds are considered as the training set and the remaining fold as the test set. This process continues until all folds have been considered once as a training set and once as a test set. This approach was used since the number of samples was too low for the classically portioned separate training and test sets. The hyperparameters of each prediction model were tuned through a 3-fold cross-validation, using a grid or random search algorithm, depending on the number of hyperparameters and the computational limitations. We selected hyperparameters that generated lower average cross-validation root mean square error (RMSE) values. Using the tuned hyperparameters, training mean absolute percent error (MAPE) was calculated based on all samples, whereas a 10-fold cross-validation with 30 replications was adopted to calculate the test MAPE. The lower the MAPE, the better the model performs, and the closer the training and test MAPE are, the less the model is prone to overfitting. RMSE and MAPE are defined as follows:


$$\begin{array}{l} RMSE=\;\sum_{i=1}^{n}\sqrt{{\left(\hat{y_{i}}-y_{i}\right)}^{2}}\\ MAPE=\frac{1}{n}\sum_{i=1}^{n}{\frac{\left|\hat{y_{i}}-y_{i}\right|}{y_{i}}}^{*}100 \end{array}$$

where *y*_*i*_, $\hat{y_{i}},$ and *n* define the measured relative abundance of *i*^*th*^ OTU, the predicted value of relative abundance of *i*^*th*^ OTU, and the number of samples in the set (training set or each fold in the cross-validation), respectively.

### Model Descriptions

The linear (multiple linear regression) model also known as least square regression predicts a line that is the best possible fit to the data points, for which the formula is as follows:


$$\begin{array}{l} {\hat{y}}_{i}=\;{\hat{\beta }}_{0}+\;{\hat{\beta }}_{1}x_{i1}+\;{\hat{\beta }}_{2}x_{i2}+\ldots +\;{\hat{\beta }}_{P}x_{iP}\;\\ \hat{Y}=X\hat{\beta } \end{array}$$

$\hat{\beta }\;$matrix is estimated using the least squares method (Brown, 2009). Hyperparameter tuning is not defined for this prediction model since it does not have one.

Support vector machine (SVM) regression uses a linear kernel to map the feature space (P dimension) into a higher dimension (m), followed by the construction of a linear model in the new feature space, which is used for predicting a data point.

This linear model can be shown as follows:


$$\begin{array}{l} f\left(x,\;w\right)=\;\overset{m}{\underset{j=1}{\sum }}w_{j}g_{j}\left(x\right)+b \end{array}$$

$$\begin{array}{l} g_{i}\left(x\right):set\;of\;linear\;or\;nonlinear\;transformation\;\left(here\;g\left(x\right)=x\right),\\ b\;is\;the\;bias\;term\;that\;can\;be\;ignored\;by\;centering\;the\;data\; \end{array}$$

The SVM regression performs linear regression in high-dimension feature space using an insensitive loss function and simultaneously reduces model complexity by minimizing ||*w*||^2^. This can be achieved by introducing nonnegative slack variables to measure the deviation of training samples outside the defined insensitive zone. Thus, SVM regression is formulated to minimize the penalty term that controls how trade-offs are tolerated (Wang and Hu, 2005). The main difference between a linear SVR and linear regression is that SVR uses only a subset of the data, ignoring the points close to the model's prediction, and SVR's optimization function is independent of the dimensionality of the feature space (Kleynhans et al., 2017).

A decision tree is constructed by recursive partitioning, starting from the root node (known as the first parent), each node can be split into children nodes. These nodes can then be further split, and they themselves become parent nodes of their resulting children nodes. At each split, one of the features is selected to split the samples. To split the nodes at the most informative features, an objective function is defined and optimized *via* the tree learning algorithm (Kim and Hong, 2017). From the various hyperparameters examined, we identified two hyperparameters (maximum depth of the tree and the maximum number of features) that significantly influenced model performance, and thus they were used in these analyses to identify the best node split. While valuable, decision trees are computationally expensive and prone to overfitting.

Random forest is an ensemble method that combines multiple decision trees, which can reduce the variance of decision trees. The random forest begins with many bootstrap samples extracted randomly with the replacement from the original dataset. Then a regression tree is fitted to each of the bootstrap samples (Wang et al., 2016). The ensemble prediction is calculated by averaging the predictions of all trees, producing the final prediction. For the random forest in this study, we identified three significant hyperparameters for consideration in each split by using a random search approach: number of trees (*T*), maximum depth of each tree, and maximum number of features.

### Code Availability

The code is available on GitHub (https://github.com/amini-ISU/Machine-Learning-Prediction-of-Nitrification-.git). It is written using *Python 3.7* or later, and the most used package is *sklearn*.

## Results

### Model Performance

We used a hierarchical feature selection process to reduce the number of OTUs to minimize the potential for overfitting models as described in the “Materials and methods” section. Briefly, this included retention of nitrifier OTUs only if their relative abundance met a minimum threshold (1/50th of the largest relative abundance of an OTU in that taxon) and removal of OTUs if they were highly correlated (*R*^2^ ≥ ±0.8, *p* ≤ 0.05) to each other; the most abundant correlated OTU was retained (Supplementary Tables S1, S2). The analyses included 37 nitrifier OTUs and ammonia and nitrite pool sizes for a total of 39 features. In separate analyses, we also included AOA and AOB *amoA* gene abundance data, resulting in 42 features. We then performed principal component analysis on the datasets and retained only those features that collectively explained 80% of the data variance.

We evaluated the predictive performance of linear (regression and support vector machine [SVM]) and nonlinear models (decision tree and random forest) by comparing training and test accuracy (held out cross-validation) and assessing mean absolute percent error (1-MAPE). Both the linear and nonlinear models performed comparably, with the random forest consistently performing only slightly better with the inclusion of AOB and AOA *amoA* gene abundance (Tables 1, 2), which suggests that predicting nitrification could be based on microbiome nitrifier composition data alone. Both the linear regression and SVM models performed comparably, while the poorest model was the decision tree.

**Table 1:** Machine learning model accuracy (% 1-MAPE).

	**Linear regression**		**SVM**		**Decision tree**		**Random forest**	
**Model**	**Training**	**Test**	**Training**	**Test**	**Training**	**Test**	**Training**	**Test**
Conventional bulk soil	87.4	82.3	87.9	80.5	82.0	66.5	81.6	74.5
Diversified bulk soil	67.5	54.6	75.3	68.1	69.3	53.5	74.4	62.5
Conventional rhizosphere	76.9	61.1	79.8	58.1	79.2	64.4	80.0	67.6
Diversified rhizosphere	81.2	74.9	84.2	71.4	82.1	56.4	82.2	74.2
Bulk soil	71.6	64.5	73.9	67.3	70.9	56.1	75.3	67.5
Rhizosphere	69.7	57.4	75.6	60.5	75.8	70.7	71.0	63.2
Conventional cropping system	69.3	57.6	76.0	61.7	70.3	67.9	69.6	62.8
Diversified Cropping system	71.3	63.5	74.1	65.3	70.7	62.9	75.6	66.7

*The best performing model (highest % test accuracy) is in bold. Blue, the best; Red, 2nd best; and Green, 3rd best*.

**Table 2:** Machine learning model accuracy (% 1-MAPE) with ammonia oxidizer *amoA* gene abundance.

	**Linear regression**		**SVM**		**Decision tree**		**Random forest**	
**Model**	**Training**	**Test**	**Training**	**Test**	**Training**	**Test**	**Training**	**Test**
Conventional bulk soil	87.2	81.9	87.8	83.6	78.8	71.8	82.7	77.6
Diversified bulk soil	66.7	59.1	71.0	72.1	78.8	59.2	75.5	66.2
Conventional rhizosphere	79.2	61.0	82.9	57.6	78.9	61.8	79.9	67.8
Diversified rhizosphere	79.1	74.3	84.9	69.0	87.4	65.1	84.5	76.6
Bu 00lk soil	71.3	63.5	74.1	65.3	70.7	62.9	75.6	66.7
Rhizosphere	59.4	57.6	76	61.7	70.3	67.9	69.6	62.8
Conventional cropping system	83.8	73.3	86.6	76.0	74.2	69.3	78.5	70.5
Diversified Cropping system	64.9	61.4	69.0	58.8	68.4	38.0	72.3	59.7

*The best performing model (highest % test accuracy) is in bold. Blue, the best; Red, 2nd best; and Green, 3rd best*.

### Interpretation of Feature Importance

**Figure 1:**
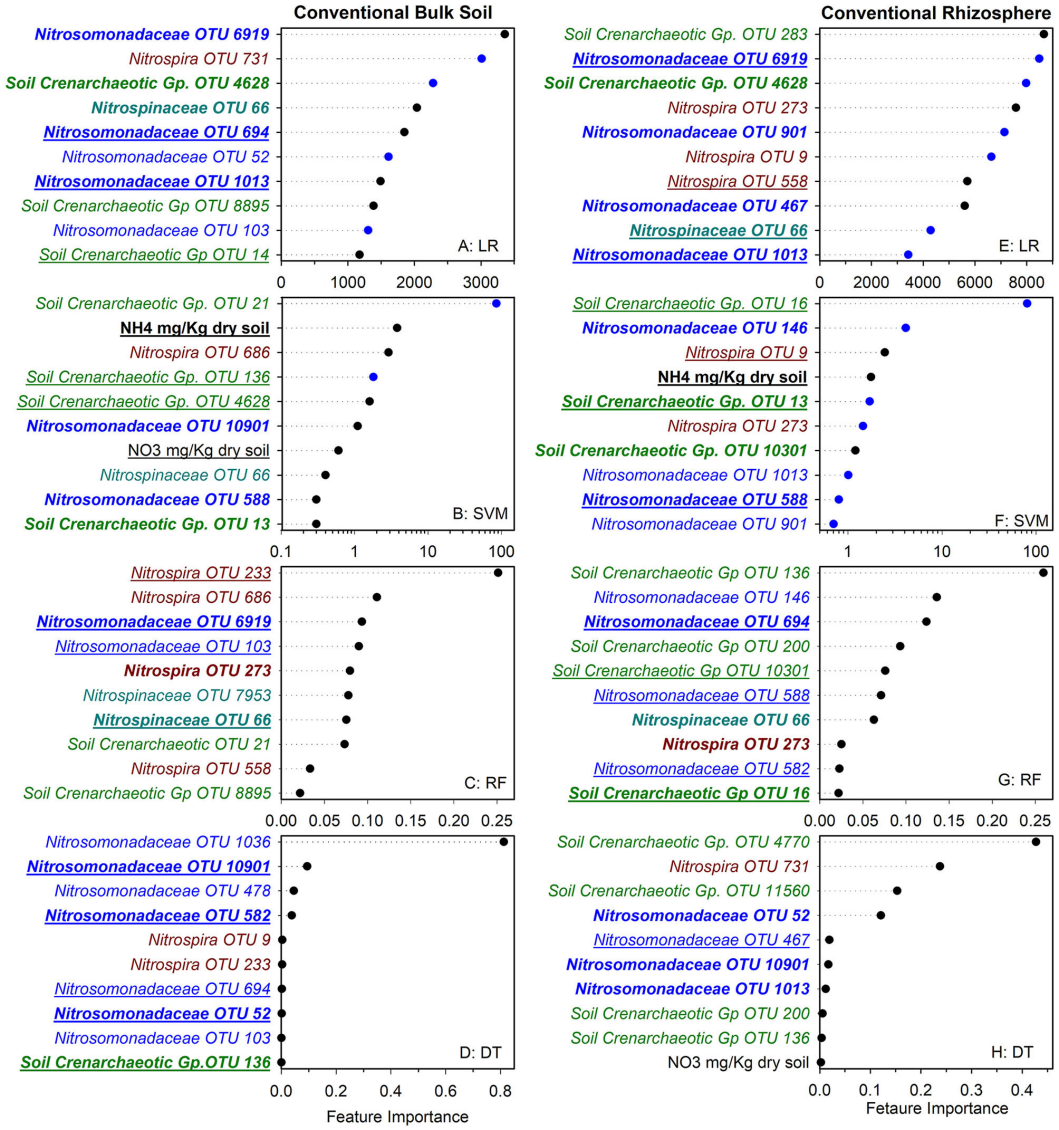
Ranks of absolute feature weights of linear **(A,B,E,F)** and nonlinear **(C,D,G,H**) models for conventional bulk soil and rhizosphere. The 10 highest ranked features in absolute feature weights are displayed from highest to lowest. In regression **(A,E)** and SVM **(B,F)**, the features and importance of OTUs that were positively associated with nitrification are represented by black dots, and those negatively associated with nitrification are shown in blue. The values for linear regression are the absolute feature importance, while for SVM, the log_10_ relative absolute feature importance. Within each model, bold OTUs indicate the same OTUs in bulk soil and rhizosphere within a cropping system, and underlined features indicate the same OTUs in bulk soil or rhizosphere between cropping systems. Random Forest **(C,G)**. Decision Tree **(D,H)**.

**Figure 2:**
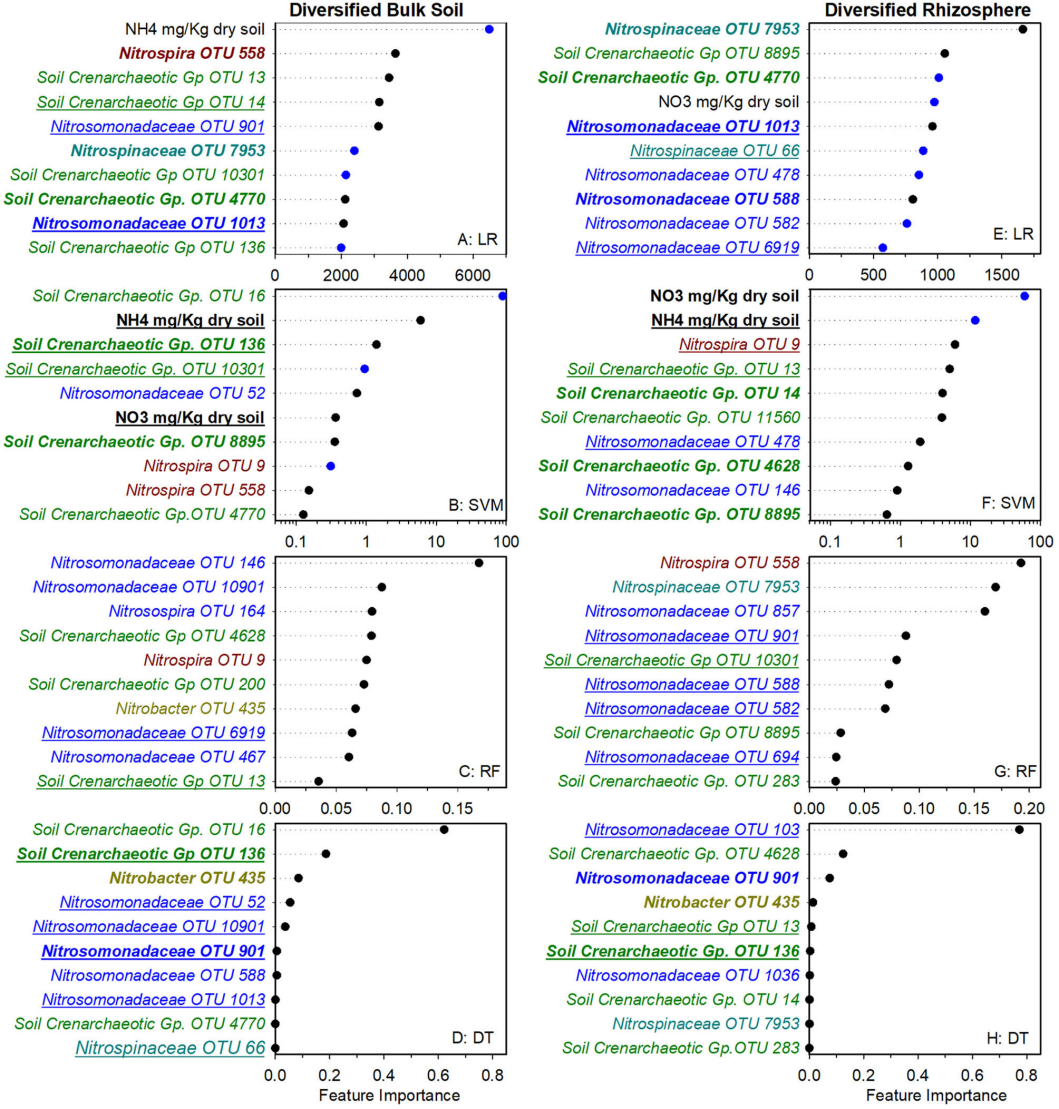
Ranks of absolute feature weights of linear **(A,B,E,F)** and nonlinear **(C,D,G,H)** models for diversified bulk soil and rhizosphere. The 10 highest ranked features of absolute feature weights are displayed from highest to lowest. In regression **(A,E)** and SVM **(B,F)**, feature importance of OTUs positively associated with nitrification are represented by black dots, and those negatively associated with nitrification are shown in blue. Values for linear regression are the absolute feature importance, while for SVM, the log_10_ relative absolute feature importance. Within each model, bold OTUs indicate the same OTUs in bulk soil and rhizosphere within a cropping system and underlined features indicate the same OTUs in bulk soil or rhizosphere between cropping systems. Random Forest **(C,G)**. Decision Tree **(D,H)**.

To identify nitrifier ecotypes unique to the rhizosphere or a cropping system, we evaluated the feature importance extracted from machine learning models. We interpreted the feature importance of linear models using the rank of absolute feature weight for each feature and by whether the signs of feature weights were predictive of potential nitrification rates, as described previously (Topçuoglu et al., 2020). While only amendable to linear models, it provides information on the sign and magnitude of each feature in predicting nitrification. Very few of the highest ranked features were shared between linear and SVM regression for each cropping system by rhizosphere/soil comparison, with diversified bulk soil having the most in common (5 out of 10) (Figures 1, 2). Only *Nitrosomonadaceae* OTU 1013 and NH${}_{4}^{+}$ pool size was common in bulk soil and rhizosphere in both cropping systems in the linear and SVM models, respectively. For each model, there were more common features within a cropping system than when comparing the soil or rhizosphere between cropping systems (Supplementary Table S3). For the nonlinear models, we evaluated ranked feature importance but lack information on whether a feature contributes positively or negatively to the model. Diversified bulk soil had the fewest (1 out of 10) while the diversified and conventional rhizospheres had 2–3 (out of 10) features in common between random forest and decision tree. The decision tree model identified soil crenarchaeotic Gp. OTU 136 was identified as an important feature in the bulk soil and rhizosphere in both cropping systems; no common OTUs were identified in the random forest model. Compared to the linear models, the nonlinear models had fewer OTUs in common within cropping systems, and between soil and rhizosphere (Figures 1, 2). Interestingly, there were no common OTUs between the diversified bulk soil and the rhizosphere (Figure 2 and Supplementary Table S3). For each cropping system and soil/rhizosphere combination, there were no common OTUs among the four machine learning models.

**Figure 3:**
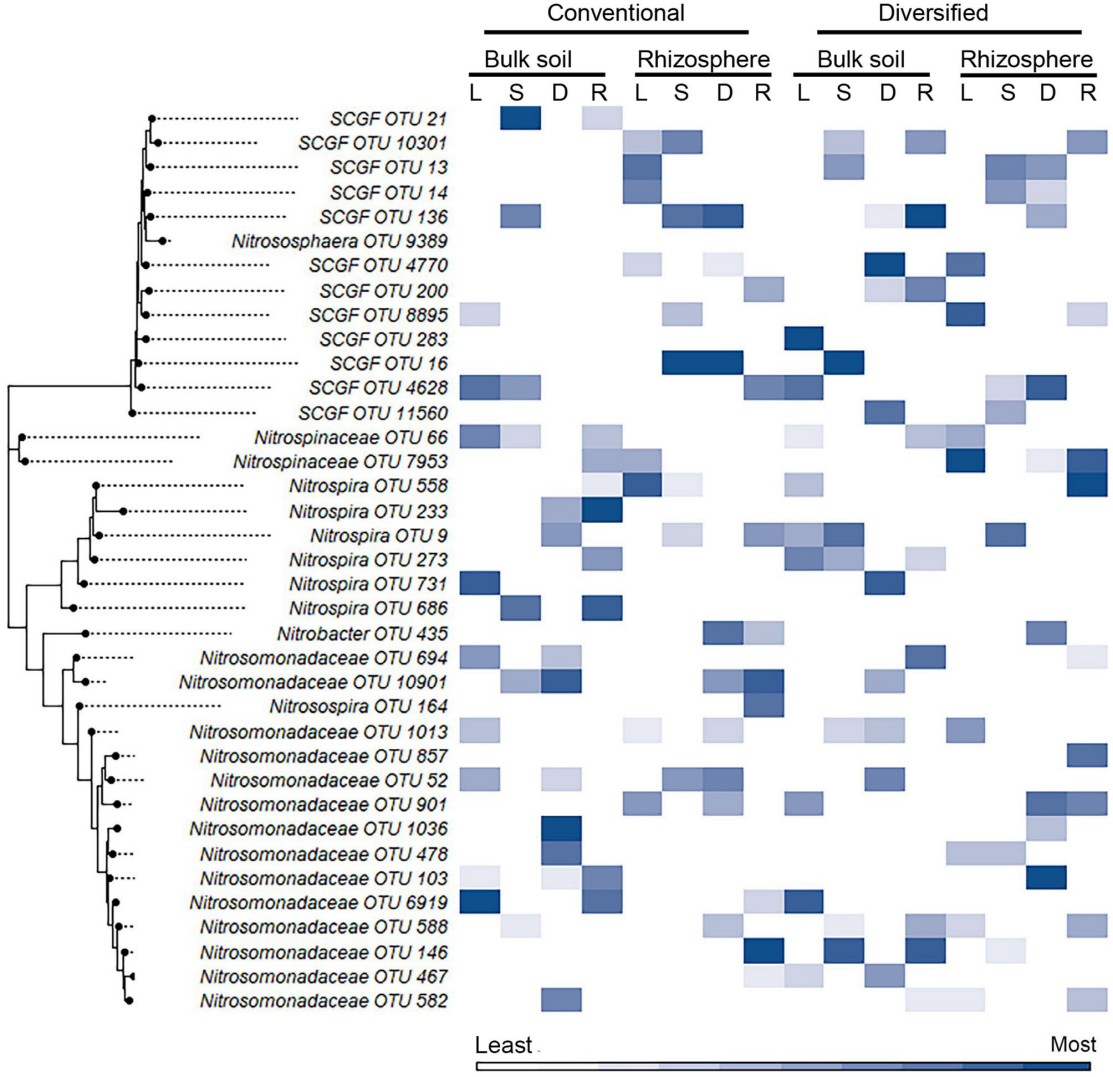
Heat map of importance of specific OTU's in linear and nonlinear models for each cropping system by habitat. OTU 16S V4 sequences were aligned using ClustalW in MEGA11, and the trees were generated using the maximum likelihood method with default parameters. Heat map color (within a column) reflects importance of the top 10 OTUs: white reflects OTUs ranked 10th or below in model importance (linear regression: L; support vector machine: S; decision tree: D; random forest: R). SCGF, soil crenarchaeotic group family.

We also explored whether linear or nonlinear models identified different nitrifier taxa as top ranked features. There were more highly ranked *Nitrosomonas* and *Nitrospirae* OTUs in the conventional soil and rhizosphere, while there were more *Thaumarchaea* in the diversified soil and rhizosphere (Figures 1, 2). Interestingly, in the bulk soil, the nitrite oxidizers that are present are often represented within the first several important features. Although we identified patterns in higher taxonomic levels between treatments, there is less consensus between individual OTUs present in the most important features. Most OTUs are identified as an important feature in at least one model across treatments but were not consistently identified in the top 10 features of a specific cropping system or in the rhizosphere. Importantly, OTU relative abundance does not predicate importance, with high abundance OTUs having both high and low importance (Supplementary Tables S4). When comparing feature importance ranks by phylogenetic relationships, there are a few discernable patterns (Figure 3). For example, the clade comprising *Nitrosomonadaceae* OTUs 6919, 586, 146, 467, and 582 where comprised of most of the highly ranked OTUs within this family. Likewise, the most highly ranked *Nitrospira* OTUs (9, 233, and 558) are within the same node (Figure 3).

**Figure 4:**
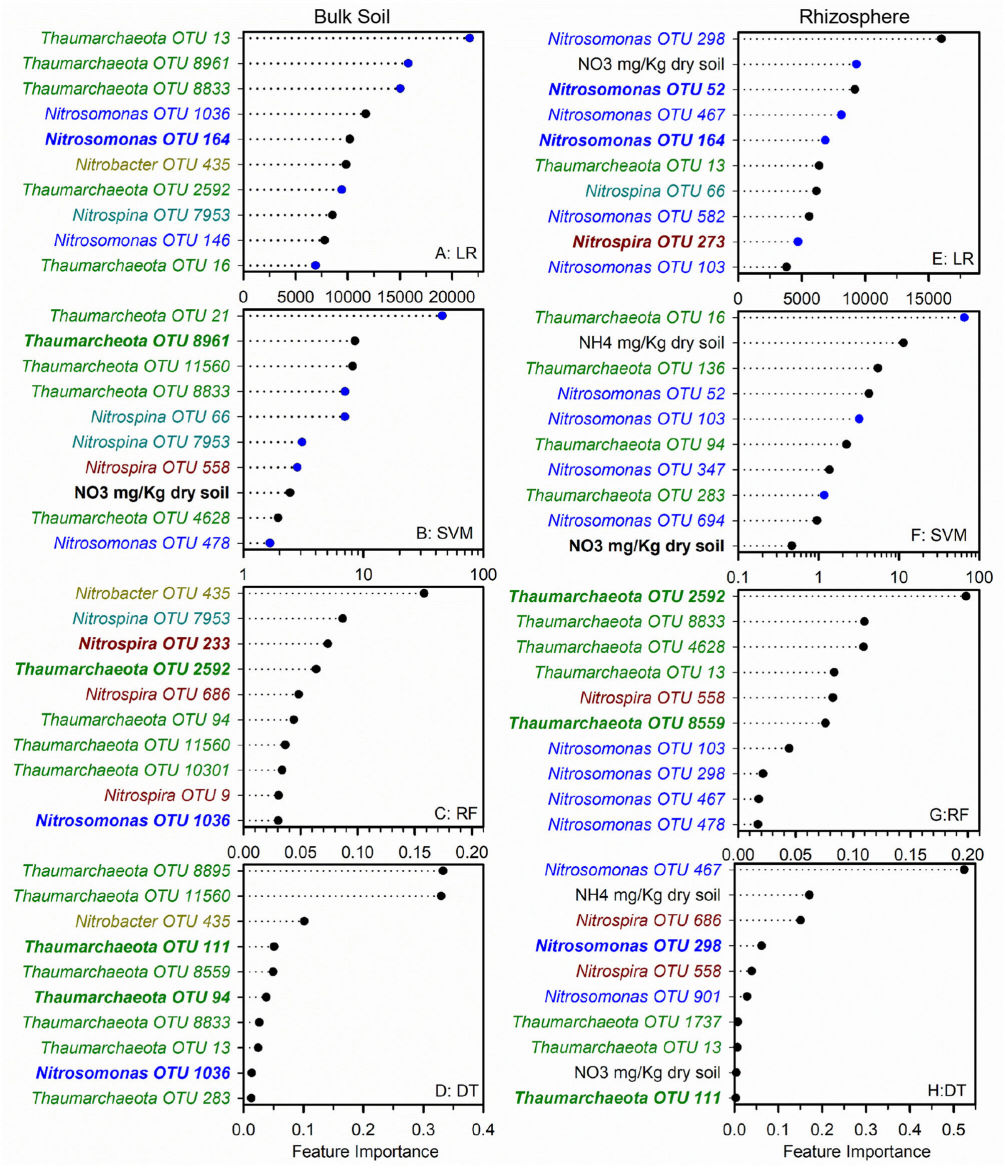
Ranks of absolute feature weights of linear **(A,B,E,F)** and nonlinear **(C,D,G,H)** models for bulk soil and rhizosphere. The 10 highest ranked features of absolute feature weights are displayed from highest to lowest. In regression **(A,E)** and SVM **(B,F)**, feature importance of OTUs positively associated with nitrification are represented by black dots, and those negatively associated with nitrification are shown in blue. The values for linear regression are the absolute feature importance, while for SVM, the log_10_ relative absolute feature importance. Within each model, bold OTUs indicate the same OTUs in bulk soil and rhizosphere. Random Forest **(C,G)**. Decision Tree **(D,H)**.

**Figure 5:**
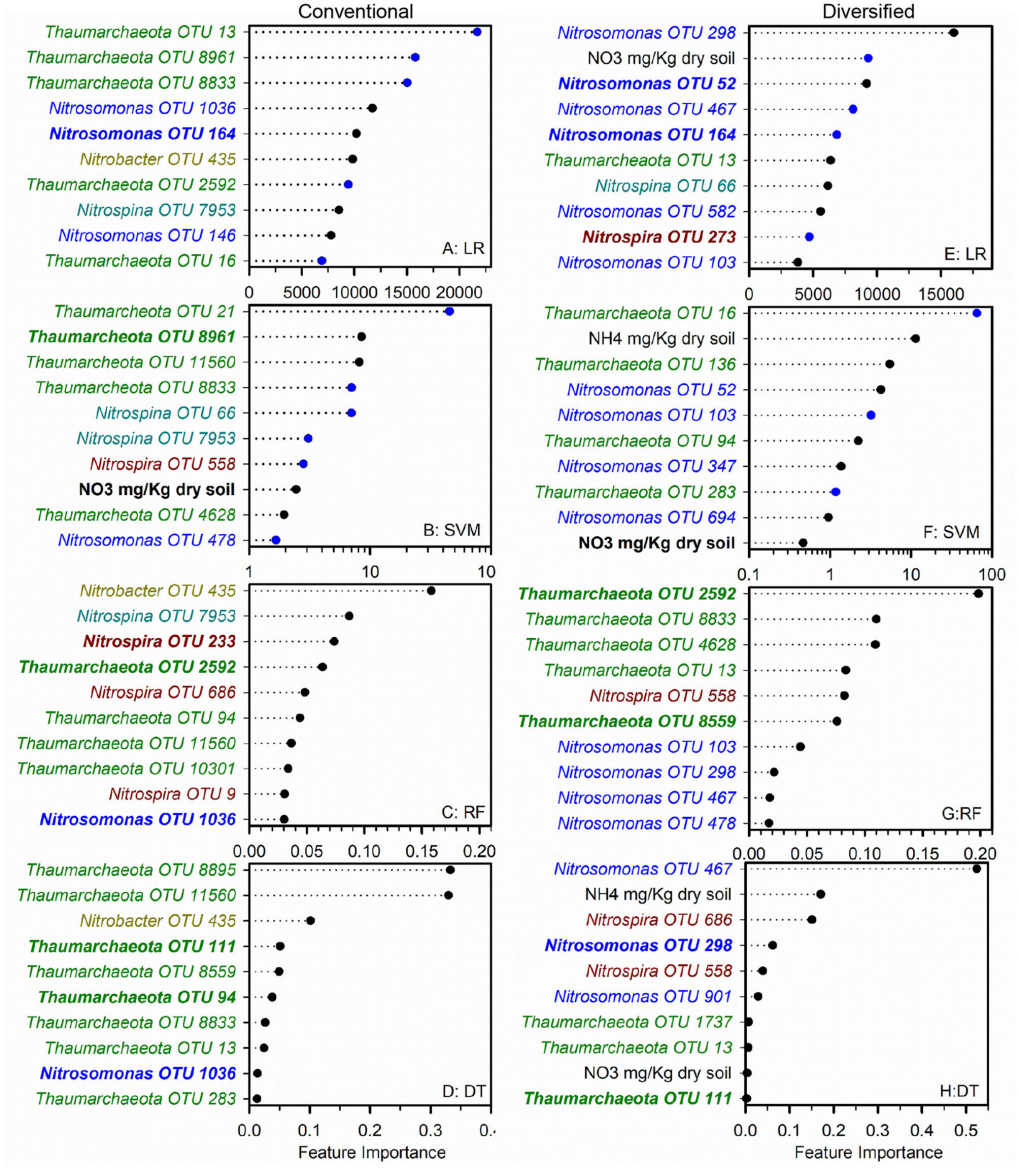
Ranks of absolute feature weights of linear **(A,B,E,F)** and nonlinear **(C,D,G,H)** models for each cropping system. The 10 highest ranked features of absolute feature weights are displayed from highest to lowest. In regression **(A,E)** and SVM **(B,F)**, feature importance of OTUs positively associated with nitrification are represented by black dots, and those negatively associated with nitrification are shown in blue. The values for linear regression are the absolute feature importance, while for SVM, the log_10_ relative absolute feature importance. Within each model, bold OTUs indicate the same OTUs in both cropping systems. Random Forest **(C,G)**. Decision Tree **(D,H)**.

**Figure 6:**
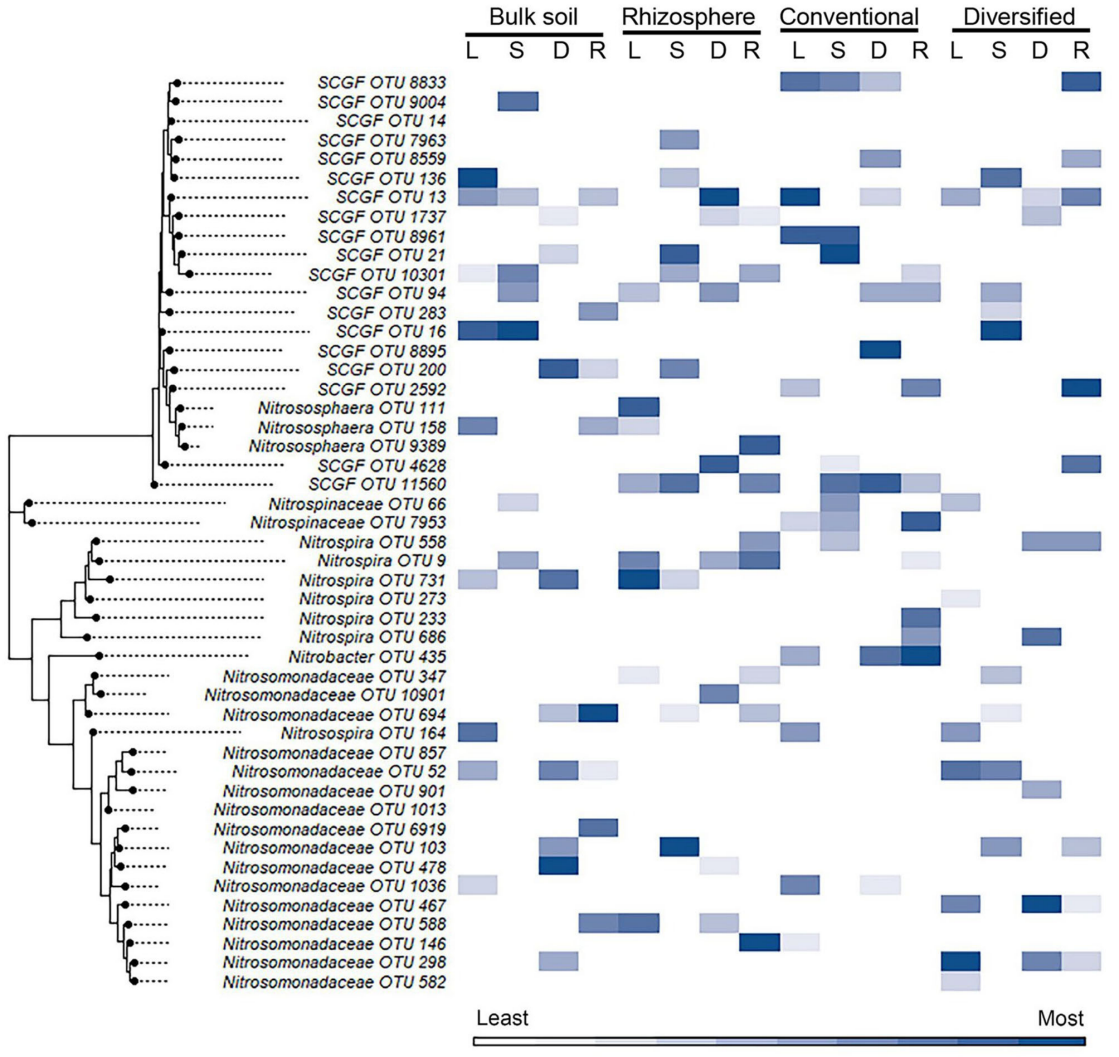
Heat map of importance of specific OTU's in linear and nonlinear models examining habitat and cropping system. OTU 16S V4 sequences were aligned using ClustalW in MEGA11, and the trees were generated using the maximum likelihood method with default parameters. Heat map color (within a column) reflects importance of the top 10 OTUs: white reflects OTUs ranked 10th or below in model importance (linear regression: L; support vector machine: S; decision tree: D; random forest: R). SCGF, soil crenarchaeotic group family.

While cropping system or rhizosphere datasets were twice as large as individual cropping system and soil/rhizosphere comparisons, the increase in dataset size did not improve model performance (Tables 1, 2). While the inclusion of ammonia-oxidizer *amoA* gene abundance greatly improved the accuracy of all models of the conventional system, it decreased the accuracy of models of the diversified system: it had little effect in bulk soil or rhizosphere. This is surprising given that there are significant correlations between nitrification and AOB *amo*A gene abundance in both conventional and diversified systems, but without a consistent pattern in the cropping system by soil/rhizosphere comparisons (Supplementary Table S5). There were no common OTUs in either the soil/rhizosphere or cropping system analyses (Figures 4, 5), and there was no relationship between feature importance and relative abundance (Supplementary Table S6). There was equal representation of *Thaumarchaeota* in the soil and rhizosphere models, with slightly more *Nitrosomonas* in soil compared to rhizosphere. Interestingly, there were more *Nitrosomonas* OTUs in the top ranked features in the diversified compared to conventional systems, while there were more nitrite-oxidizers in the conventional system. When comparing feature importance ranks by phylogenetic relationships, the clade comprised of *Thaumarchaeota* OTUs 13, 1737, 8961, 21, and 10301 were highly ranked features in the rhizosphere, and conventional system, but not the diversified system or bulk soil (Figure 6). In contrast, the diversified system had many highly ranked features within a *Nitrosomonadacae* clade comprised of OTUs 467, 588, 146, 298, and 582 (Figure 6).

### Inclusion of Ammonia-Oxidizer *amoA* Abundance in Predictive Modeling

We assessed how inclusion of ammonia-oxidizer *amo*A abundance influenced feature importance profiles in random forest given that model accuracy was similar with or without its inclusion (Tables 1, 2). Generally, the ammonia oxidizer *amo*A abundance ratio was a highly ranked (within the top 10) feature regardless of the type of comparison (Supplementary Figures S1–S3). In conventional soil, despite only slightly increasing model accuracy, inclusion of AOA/AOB abundance made it the most important feature (~42%), although it was not highly correlated with nitrification potential in of itself (Supplementary Table S5). Yet, despite the influence of including ammonia-oxidizer *amoA* abundance on random forest model accuracy of the diversified system (Table 2), AOB *amo*A abundance was not a highly ranked feature in the conventional soil. In the rhizosphere analysis, AOB *amo*A abundance became a top ranked feature despite it not being highly correlated to nitrification potential (Supplementary Table S5) and did not substantively improve model accuracy (Table 2).

## Discussion

Our results indicate that nitrification potential can be predicted from nitrifier microbiome composition data using machine learning and that the results can be as robust as traditional multivariate linear regression or correlation approaches (Herman et al., 2003; Booth et al., 2005; Tourna et al., 2008). Machine learning offers the ability to incorporate the structure of the microbial communities or a subset of community members to identify associations between community structure and a biogeochemical process (Thompson et al., 2019) or a diseased state (Sze et al., 2019; Topçuoglu et al., 2020). Traditional statistical approaches frequently rely on identifying whether a single organism or group of organisms, such as ammonia oxidizing bacteria or archaea *amo*A gene or transcript abundance (Tourna et al., 2008; Verhamme et al., 2011; Ouyang and Norton, 2020) is associated with a process/state. While much has been learned about the contributions of AOA or AOB to potential nitrification activity (Herman et al., 2003; Ouyang and Norton, 2020), less is known about the contribution of different nitrifier ecotypes to nitrification rates.

Both linear and nonlinear approaches generated results with equivalent accuracy, each providing information on the relative importance of taxonomic and nontaxonomic features to the model. The failure to identify common OTUs or clades of OTUs predictive of how management or the root influences nitrification could reflect a limitation of this approach or that each habitat harbors a unique nitrifier community with unique nitrifying potential. Despite, from a biological perspective, having relatively large sample sizes (*n* = 15–16), machine learning is most appropriate with very large datasets (often with *n* > 300). Yet, performance accuracy (test 1-MAPE %) did not improve substantially overall when doubling the sample size in the rhizosphere, bulk soil, and cropping system analyses. This highlights the potential risks associated with merging data from distinct microbial habitats (e.g., rhizosphere vs. soil or different cropping systems), particularly if the number of samples from each habitat is relatively small. Sensitivity analyses of model prediction performance could provide insight into the appropriate dataset size (Thompson et al., 2019). Additionally, by including only the taxa capable of ammonia or nitrite oxidation, we may have excluded community members who could provide additional insight. While it would have been desirable to include other taxa as additional features, the large number of OTUs (~3,000) dwarfed the number of samples, increasing the risk of overfitting the model. It is important to note that our analysis was based on measurements of the potential for nitrification and microbiome profiles using samples from a single time point (Bay et al., 2021). Sampling more frequently, more extensively, and/or at a finer granular scale may provide a more robust classification and improve accuracy. Additionally, deep learning methods (Oh and Zhang, 2020; Reiman et al., 2021) could be explored, but our dataset may suffer from too few samples for such analyses. In our case, few-shot learning (FSL) may be more appropriate, as it relies less on large sample sizes (Sung et al., 2018).

Ammonia and nitrate pool sizes have been used for predicting nitrification and/or the growth of ammonia oxidizers (Herman et al., 2003; Verhamme et al., 2011), using standard statistical techniques, such as stepwise linear regression. Here, inorganic N pool sizes were more often identified as a top ranked feature in linear than in nonlinear models, with NH${}_{4}^{+}$ pool size being more highly ranked in SVM models. There was no single taxonomic group whose feature rankings were consistently higher than other taxonomic groups. For example, *Nitrosomonas* and *Thaumarchaea* generally represented a similar frequency of top ranked features, with some variation between specific analyses. What did differ was the representation of specific OTUs (potential ecotypes) between specific cropping systems soil and rhizosphere communities. By identifying common highly ranked OTUs and considering whether those OTUs represent one or more co-correlated OTUs, we can begin to develop hypotheses about relationships between these features and nitrification, and how the plant or management influences those relationships.

We anticipated that the gradients in physiochemical and microbial properties present in different cropping systems and between the rhizosphere and bulk soil would provide a means to determine if specific ecotypes contribute to nitrification (Smith et al., 2014; Hink et al., 2018). Our analyses revealed some relationships between feature importance and phylogenetic similarity in some of the comparisons. For example, specific *Nitrosomonadaceae* and *Thaumarchaeota* clades comprised large numbers of highly ranked features in multiple models for specific cropping systems or for soil/rhizosphere. Likewise, there was the absence of certain *Nitrospira* lineages in both conventional and diversified rhizospheres. Whether these patterns reflect the identification of specific ecotypes needs to be determined experimentally in future studies assessing nitrification rates by these specific lineages.

In general, random forest outperformed the linear models and decision trees, without or with the inclusion of ammonia oxidizer *amoA* gene abundance data. Random forest is relatively easy to perform, but interpretability of the influence of selected features on the nitrification process is more difficult due to the lack of insight into whether a feature positively or negatively influences nitrification. As we do not know *a priori* which set of ecotypes drives changes in nitrification, the selection of features from a group of models may be better than from one method alone. Our results suggest that inclusion of the time-consuming (and costly qPCR) ammonia-oxidizer *amoA* abundance data is not essential. Yet, although model performance did not change appreciably when it was included, it did dramatically influence the identity and position of highly ranked features, providing different perspectives on taxa that are predictive of nitrification. For example, in conventional bulk soil, the AOA/AOB *amo*A ratio was the most highly ranked feature (42%), and only 25% of the same features (OTUs) were highly ranked in both analyses.

Our analysis provides insight into the suitability of using a subset of 16S rRNA microbiome composition data to model a biogeochemical process mediated by a relatively narrow group of organisms. Our findings highlight the need to compare different machine learning models and that the identification of ecotypes predictive of nitrification is model dependent, providing a basis for constructing hypotheses to assess relationships between nitrifier ecotypes on nitrogen cycling dynamics. This may entail exploring the relative metabolic activity of different nitrifying clades in each cropping system and in the rhizosphere, since bulk soil does not adequately reflect nitrification and nitrifier community assembly near plant roots.

## Data Availability Statement

The datasets presented in this study can be found in online repositories. The names of the repository/repositories and accession number(s) can be found below: https://www.ncbi.nlm.nih.gov/, PRJNA686799.

## Author Contributions

CL assembled datasets for machine learning analyses, including feature selection, performed all analyses of machine learning models and feature predictions, and wrote the first draft of the manuscript. FA performed all machine learning analyses and edited the manuscript drafts. GH and LH designed and oversaw research, as well as contributed to writing the manuscript. All authors contributed to the article and approved the submitted version.

## Funding

This study was supported by the USDA AFRI grant program (grant 2014-67019-21628), the Iowa Agriculture and Home Economics Experiment Station, and the Plant Sciences Institute at Iowa State University.

## Conflict of Interest

The authors declare that the research was conducted in the absence of any commercial or financial relationships that could be construed as a potential conflict of interest.

## Publisher's Note

All claims expressed in this article are solely those of the authors and do not necessarily represent those of their affiliated organizations, or those of the publisher, the editors and the reviewers. Any product that may be evaluated in this article, or claim that may be made by its manufacturer, is not guaranteed or endorsed by the publisher.
