# Supplementary material for: Machine Learning Prediction of Nitrification From Ammonia- and Nitrite-Oxidizer Community Structure
Source: Front Microbiol. 2022 Jul 11;13:899565. doi: 10.3389/fmicb.2022.899565 (PMC9309558; doi:10.3389/fmicb.2022.899565)
Supplement: Supplementary file 1 [file Data_Sheet_1.docx]

**Supplemental Figures and Tables**

**Table S1**. OTUs removed due to high co-correlation (right column) and corresponding retained OTUs (left column) for developing soil/rhizosphere by cropping system models.

| Retained OTU | Co-correlated OTU(s) |
| --- | --- |
| Soil Crenarchaeotic Gp. Fam. OTU 13 | Soil Crenarchaeotic Gp. Fam OTU 80 |
|  | *Nitrosomonadaceae* Unc. Sp.OTU 1677 |
|  | Soil Crenarchaeotic Gp. Fam OTU 2965 |
|  | *Nitrososphaera* Unc. Sp.OTU 158 |
|  | Soil Crenarchaeotic OTU 7963 |
|  | *Nitrospinaceae* Sp.OTU 1906 |
|  | Soil Crenarchaeotic Gp. Fam. OTU 8961 |
|  | Soil Crenarchaeotic Gp. Fam. OTU 1737 |
|  | Soil Crenarchaeotic Gp. Fam. OTU 8833 |
|  | Soil Crenarchaeotic Gp. Fam.OTU 9004 |
| Soil Crenarchaeotic Gp. Fam. OTU 8895 | Soil Crenarchaeotic Gp. Fam. OTU 94 |
| *Nitrosomonadaceae* OTU 52 | *Nitrosomonadaceae* OTU 298 |
| Soil Crenarchaeotic Gp. Fam. OTU 283 | Soil Crenarchaeotic Gp. Fam. OTU 8559 |
| Soil Crenarchaeotic Gp. Fam. OTU 16 | *Nitrososphaera* OTU 111 |
| Soil Crenarchaeotic Gp. Fam. OTU 200 | Soil Crenarchaeotic Gp. Fam. OTU 2592 |
| *Nitrosomonadaceae* Sp. OTU 103 | *Nitrosomonadaceae* OTU 347 |

| Retained OTU | Co-correlated OTU(s) | |
| --- | --- | --- |
| Bulk Soil and Rhizosphere Models | | |
| Soil Crenarchaeotic Gp. Fam. OTU 136 | | Soil Crenarchaeotic OTU 4770 |
| *Nitrososphaera* Unc. Sp.OTU 158 | | *Nitrosomonadaceae* Unc. Sp.OTU 1677 |
|  | | Soil Crenarchaeotic Gp. Fam OTU 80 |
| Soil Crenarchaeotic Gp. Fam. OTU 13 | | *Nitrospinaceae* UGUSp.OTU 1906 |
|  | | Soil Crenarchaeotic Gp. Fam OTU 2965 |
|  | | Soil Crenarchaeotic Gp. Fam. OTU 8961 |
| Soil Crenarchaeotic Gp. OTU 10301 | | Soil Crenarchaeotic Gp. Fam. OTU 8833 |
| Soil Crenarchaeotic Gp. Fam. OTU 16 | | Soil Crenarchaeotic Gp. Fam. OTU 8559 |
| *Nitrosomonadaceae* OTU 6919 | | *Nitrospira* Unc. Sp.OTU 273 |
|  | | *Nitrosomonadaceae* Unc. Sp.OTU 467 |
| *Nitrososphaera* OTU 111 | | Soil Crenarchaeotic Gp. Fam. OTU 2592 |
|  | | *Nitrosomonadaceae* OTU 901 |
|  | |  |
| Conventional and Diversified Models | | |
| *Nitrososphaera* Unc. Sp.OTU 158 | | Soil Crenarchaeotic OTU 7963 |
| Soil Crenarchaeotic Gp. Fam. OTU 13 | | Soil Crenarchaeotic Gp. Fam OTU 80 |
|  | | *Nitrosomonadaceae* Unc. Sp.OTU 1677 |
|  | | *Nitrosomonadaceae* OTU 588 |
| *Nitrospinaceae* OTU 66 | | *Nitrospinaceae* UGUSp.OTU 1906 |
| Soil Crenarchaeotic Gp. Fam. OTU 16 | | *Nitrospira* Unc. Sp.OTU 731 |
| Soil Crenarchaeotic Gp. OTU 10301 | | Soil Crenarchaeotic Gp. Fam OTU 2965 |
| Soil Crenarchaeotic Gp. Fam. OTU 136 | | Soil Crenarchaeotic OTU 4770 |
| Soil Crenarchaeotic Gp. Fam. OTU 14 | | Soil Crenarchaeotic Gp. Fam.OTU 9004 |

**Table S2**. OTUs removed due to high co-correlation (right column) and corresponding retained OTUs (left column) for developing soil/rhizosphere and cropping system models.

| **Table S3.** Proportion of common features among the 12 highest ranked features | | | | |
| --- | --- | --- | --- | --- |
|  | LR | SVM | DT | RF |
| Soil: Conventional vs Diversified | 0.25 | 0.42 | 0.50 | 0.42 |
| Conventional: soil vs rhizosphere | 0.42 | 0.42 | 0.42 | 0.33 |
| Rhizosphere: Conventional vs Diversified | 0.33 | 0.42 | 0.25 | 0.42 |
| Diversified: Soil vs rhizosphere | 0.42 | 0.50 | 0.25 | 0.00 |

**Table S4.** Relative abundance of OTUs used in machine learning models examining bulk soil and rhizospheres in conventional and diversified cropping systems. Values in bold reflect OTUs that were a top ten feature in at least one model. Conv= Conventional; Div= Diversified.

|  | Relative abundance (%) | | | | | | |
| --- | --- | --- | --- | --- | --- | --- | --- |
|  | Bulk soil | | |  | Rhizosphere | | |
| **Feature** | **Conv** |  | **Div** |  | **Conv** |  | **Div** |
| *Nitrososphaera* Unc. Sp.OTU 9389 | 0.78% |  | 0.33% |  | 0.51% |  | 0.28% |
| Soil Crenarchaeotic Gp. Fam. OTU 136 | **0.10%** |  | **0.02%** |  | **0.05%** |  | **0.01%** |
| Soil Crenarchaeotic Gp. Fam. OTU 10301 | 0.26% |  | **0.48%** |  | **0.23%** |  | **0.48%** |
| Soil Crenarchaeotic Gp. Fam. OTU 13 | **1.01%** |  | **1.76%** |  | **0.47%** |  | **1.35%** |
| Soil Crenarchaeotic Gp. Fam. OTU 21 | **1.30%** |  | 1.53% |  | 0.79% |  | 1.09% |
| Soil Crenarchaeotic Gp. Fam. OTU 283 | 0.03% |  | 0.10% |  | **0.02%** |  | **0.08%** |
| Soil Crenarchaeotic Gp. Fam. OTU 8895 | **0.17%** |  | **0.08%** |  | 0.15% |  | **0.09%** |
| Soil Crenarchaeotic Gp. Fam. OTU 16 | 2.11% |  | **0.73%** |  | **1.23%** |  | 0.39% |
| Soil Crenarchaeotic Gp. Fam. OTU 200 | 0.30% |  | **0.17%** |  | **0.17%** |  | 0.13% |
| Soil Crenarchaeotic Gp. Fam. OTU 11560 | 0.20% |  | 0.05% |  | **0.13%** |  | **0.03%** |
| Soil Crenarchaeotic Gp. Fam. OTU 4628 | **0.92%** |  | **0.46%** |  | **0.51%** |  | **0.25%** |
| Soil Crenarchaeotic Gp. Fam. OTU 14 | 2.17% |  | **1.10%** |  | **1.23%** |  | **0.93%** |
| Soil Crenarchaeotic Gp. Fam. OTU 4770 | 0.55% |  | **0.32%** |  | **0.34%** |  | **0.19%** |
| *Nitrospira* OTU 9 | **1.08%** |  | **0.97%** |  | **0.58%** |  | **0.80%** |
| *Nitrospira* OTU 731 | **0.08%** |  | 0.02% |  | **0.06%** |  | 0.02% |
| *Nitrospira* OTU 233 | **0.13%** |  | 0.03% |  | 0.07% |  | 0.01% |
| *Nitrospira* OTU 558 | **0.04%** |  | **0.05%** |  | **0.02%** |  | **0.02%** |
| *Nitrospira* OTU 273 | **0.02%** |  | 0.14% |  | **0.00%** |  | 0.12% |
| *Nitrospira* OTU 686 | **0.02%** |  | 0.05% |  | 0.01% |  | 0.03% |
| *Nitrobacter* OTU 435 | 0.03% |  | **0.01%** |  | 0.05% |  | **0.04%** |
| *Nitrosospira* Unc. Sp.OTU 164 | 0.17% |  | **0.06%** |  | 0.04% |  | 0.02% |
| *Nitrosomonadaceae* OTU 146 | 0.08% |  | **0.09%** |  | **0.05%** |  | **0.08%** |
| *Nitrosomonadaceae* OTU 582 | **0.04%** |  | 0.05% |  | **0.03%** |  | **0.04%** |
| *Nitrosomonadaceae* OTU 52 | **0.39%** |  | **0.31%** |  | **0.16%** |  | 0.17% |
| *Nitrosomonadaceae* OTU 857 | 0.01% |  | 0.02% |  | 0.01% |  | **0.01%** |
| *Nitrosomonadaceae* OTU 1013 | **0.01%** |  | **0.02%** |  | **0.01%** |  | **0.02%** |
| *Nitrosomonadaceae* OTU 103 | **0.03%** |  | 0.11% |  | 0.04% |  | **0.18%** |
| *Nitrosomonadaceae* OTU 478 | **0.01%** |  | 0.04% |  | 0.01% |  | **0.05%** |
| *Nitrosomonadaceae* OTU 10901 | **0.08%** |  | **0.05%** |  | **0.03%** |  | 0.04% |
| *Nitrosomonadaceae* OTU 6919 | **0.01%** |  | **0.06%** |  | **0.01%** |  | **0.10%** |
| *Nitrosomonadaceae* OTU 694 | **0.02%** |  | 0.04% |  | **0.02%** |  | **0.04%** |
| *Nitrosomonadaceae* OTU 467 | 0.01% |  | **0.04%** |  | **0.00%** |  | 0.03% |
| *Nitrosomonadaceae* OTU 1036 | **0.01%** |  | 0.01% |  | 0.02% |  | **0.03%** |
| *Nitrosomonadaceae* OTU 901 | 0.04% |  | **0.02%** |  | **0.03%** |  | **0.00%** |
| *Nitrosomonadaceae* OTU 588 | **0.06%** |  | **0.04%** |  | **0.03%** |  | **0.03%** |
| *Nitrospinaceae* OTU 7953 | **0.01%** |  | **0.02%** |  | 0.00% |  | **0.01%** |
| *Nitrospinaceae* OTU 66 | **0.10%** |  | **0.19%** |  | **0.03%** |  | **0.05%** |

| **Table S5.** Pairwise correlations between nitrification potential and ammonia-oxidizer *amo*A gene abundance. Values in bold are significantly different based on an ANOVA. | | | | | | | |
| --- | --- | --- | --- | --- | --- | --- | --- |
|  |  | AOA | | AOB | | AOA/AOB | |
|  |  | R^2^ | p-value | R^2^ | p-value | R^2^ | p-value |
| Conventional Soil |  | 0.02 | 0.95 | 0.65 | **0.008** | 0.65 | **0.008** |
| Conventional Rhizosphere |  | -0.10 | 0.70 | -0.13 | 0.63 | -0.05 | 0.86 |
| Diversified Soil |  | 0.32 | 0.25 | 0.15 | 0.59 | 0.38 | 0.16 |
| Diversified Rhizosphere |  | .02 | 0.94 | -0.54 | **0.038** | 0.76 | **0.001** |
|  |  |  |  |  |  |  |  |
| Bulk Soil |  | 0.13 | 0.48 | 0.26 | 0.16 | 0.49 | **0.006** |
| Rhizosphere |  | 0.11 | .56 | 0.27 | 0.14 | -0.11 | 0.56 |
|  |  |  |  |  |  |  |  |
| Conventional |  | 0.09 | 0.63 | 0.43 | **0.017** | 0.14 | 0.44 |
| Diversified |  | -0.02 | 0.90 | 0.46 | **0.009** | -0.05 | 0.79 |

**Table S6.** Relative Abundance of OTUs used in machine learning models examining cropping systems and the rhizosphere effect. Values in bold reflect OTUs that were in the top ten features in at least one model.

|  | **Relative Abundance (%)** | | | | | | |
| --- | --- | --- | --- | --- | --- | --- | --- |
| **Feature** | **Bulk Soil** |  | **Rhizosphere** |  | **Conventional** |  | **Diversified** |
| *Nitrososphaera* Unc. Sp.OTU 9389 | 0.06% |  | **0.03%** |  | 0.08% |  | **0.02%** |
| *Nitrososphaera* Unc. Sp.OTU 111 | 0.20% |  | **0.12%** |  | 0.28% |  | **0.04%** |
| *Nitrososphaera* Unc. Sp.OTU 158 | **0.29%** |  | **0.11%** |  | 0.20% |  | 0.20% |
| Soil Crenarchaeotic Gp. Fam. OTU 136 | **0.55%** |  | **0.40%** |  | 0.64% |  | **0.31%** |
| Soil Crenarchaeotic Gp. Fam. OTU 7963 | 0.32% |  | **0.24%** |  | 0.22% |  | 0.34% |
| Soil Crenarchaeotic Gp. Fam. OTU 2592 | 0.18% |  | 0.10% |  | **0.23%** |  | **0.06%** |
| Soil Crenarchaeotic Gp. Fam.OTU 10301 | **0.37%** |  | **0.35%** |  | **0.25%** |  | 0.48% |
| Soil Crenarchaeotic Gp. Fam. OTU 13 | **1.38%** |  | **0.91%** |  | **0.74%** |  | **1.56%** |
| Soil Crenarchaeotic Gp. Fam. OTU 21 | **1.42%** |  | **0.94%** |  | **1.04%** |  | 1.31% |
| Soil Crenarchaeotic Gp. Fam. OTU 283 | **0.06%** |  | 0.05% |  | **0.03%** |  | **0.09%** |
| Soil Crenarchaeotic Gp. Fam. OTU 14 | 1.63% |  | 1.08% |  | 1.70% |  | 1.01% |
| Soil Crenarchaeotic Gp. Fam. OTU 8895 | 0.13% |  | 0.12% |  | **0.16%** |  | 0.08% |
| Soil Crenarchaeotic Gp. Fam. OTU 16 | **1.42%** |  | 0.81% |  | **1.67%** |  | **0.56%** |
| Soil Crenarchaeotic Gp. Fam. OTU 200 | **0.24%** |  | **0.15%** |  | 0.23% |  | 0.15% |
| Soil Crenarchaeotic Gp. Fam. OTU 11560 | **0.13%** |  | **0.08%** |  | **0.17%** |  | 0.04% |
| Soil Crenarchaeotic Gp. Fam. OTU 1737 | **0.20%** |  | **0.11%** |  | 0.22% |  | **0.09%** |
| Soil Crenarchaeotic Gp. Fam. OTU 8961 | 0.08% |  | 0.07% |  | **0.03%** |  | 0.11% |
| Soil Crenarchaeotic Gp. Fam. OTU 8559 | 0.09% |  | 0.05% |  | **0.11%** |  | **0.03%** |
| Soil Crenarchaeotic Gp. Fam. OTU 4628 | 0.69% |  | **0.38%** |  | **0.72%** |  | **0.35%** |
| Soil Crenarchaeotic Gp. Fam. OTU 9004 | **0.30%** |  | **0.21%** |  | 0.32% |  | 0.20% |
| Soil Crenarchaeotic Gp. Fam. OTU 94 | **0.34%** |  | **0.18%** |  | **0.28%** |  | **0.23%** |
| Soil Crenarchaeotic Gp. Fam. OTU 8833 | 0.06% |  | 0.04% |  | **0.04%** |  | **0.06%** |
| *Nitrospira* OTU 9 | **1.03%** |  | **0.69%** |  | **0.83%** |  | 0.88% |
| *Nitrospira* OTU 731 | **0.05%** |  | **0.04%** |  | 0.07% |  | 0.02% |
| *Nitrospira* OTU 233 | 0.08% |  | 0.04% |  | **0.10%** |  | 0.02% |
| *Nitrospira* OTU 558 | 0.04% |  | **0.02%** |  | **0.03%** |  | **0.04%** |
| *Nitrospira* OTU 273 | 0.08% |  | 0.06% |  | 0.01% |  | **0.13%** |
| *Nitrospira* OTU 686 | 0.03% |  | 0.02% |  | **0.01%** |  | **0.04%** |
| *Nitrobacter* OTU 435 | 0.02% |  | 0.05% |  | **0.04%** |  | 0.03% |
| *Nitrosospira* OTU 164 | **0.11%** |  | 0.03% |  | **0.11%** |  | **0.04%** |
| *Nitrosomonadaceae* OTU 146 | 0.08% |  | **0.06%** |  | **0.06%** |  | 0.08% |
| *Nitrosomonadaceae* OTU 582 | 0.04% |  | 0.03% |  | 0.03% |  | **0.04%** |
| *Nitrosomonadaceae* OTU 52 | **0.35%** |  | 0.16% |  | 0.28% |  | **0.24%** |
| *Nitrosomonadaceae* OTU 857 | 0.02% |  | **0.01%** |  | 0.01% |  | 0.02% |
| *Nitrosomonadaceae* OTU 1013 | 0.02% |  | 0.01% |  | 0.01% |  | 0.02% |
| *Nitrosomonadaceae* OTU 103 | **0.07%** |  | **0.11%** |  | 0.04% |  | **0.15%** |
| *Nitrosomonadaceae* OTU 478 | **0.02%** |  | **0.03%** |  | **0.01%** |  | **0.04%** |
| *Nitrosomonadaceae* OTU 10901 | 0.06% |  | **0.03%** |  | 0.05% |  | 0.04% |
| *Nitrosomonadaceae* OTU 6919 | **0.03%** |  | 0.06% |  | 0.01% |  | 0.08% |
| *Nitrosomonadaceae* OTU 347 | 0.06% |  | **0.07%** |  | 0.06% |  | **0.08%** |
| *Nitrosomonadaceae* OTU 694 | **0.03%** |  | **0.03%** |  | 0.02% |  | **0.04%** |
| *Nitrosomonadaceae* OTU 467 | 0.02% |  | 0.02% |  | 0.01% |  | **0.03%** |
| *Nitrosomonadaceae* OTU 1036 | **0.01%** |  | 0.02% |  | **0.02%** |  | 0.02% |
| *Nitrosomonadaceae* OTU 901 | 0.03% |  | 0.02% |  | 0.03% |  | **0.01%** |
| *Nitrosomonadaceae* OTU 298 | **0.08%** |  | 0.06% |  | 0.06% |  | **0.08%** |
| *Nitrosomonadaceae* OTU 588 | 0.05% |  | **0.03%** |  | 0.05% |  | 0.04% |
| *Nitrospinaceae* OTU 66 | **0.15%** |  | 0.04% |  | **0.07%** |  | **0.12%** |
| *Nitrospinaceae* OTU 7953 | 0.01% |  | 0.01% |  | **0.01%** |  | 0.01% |


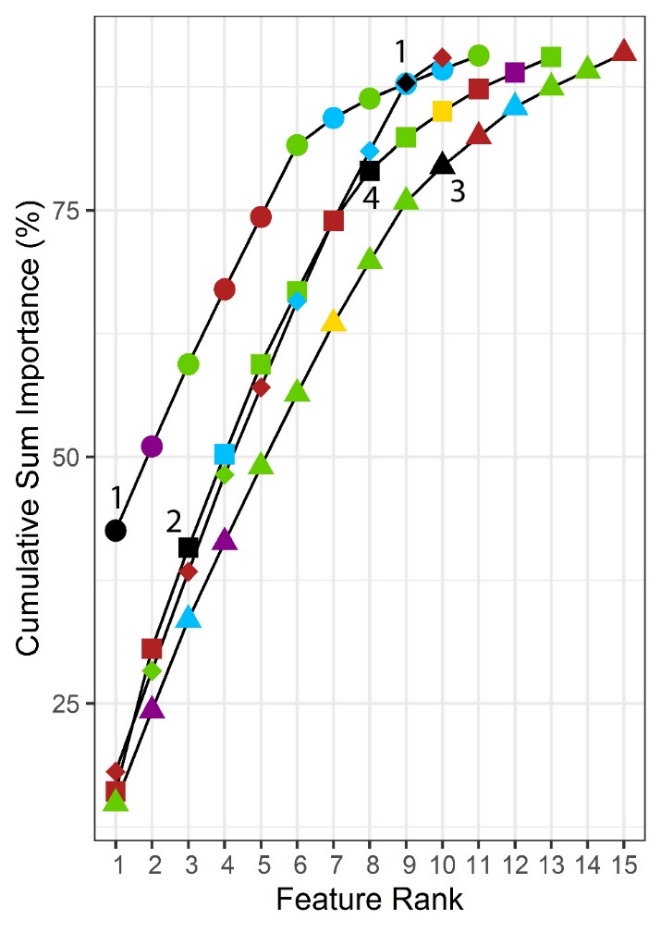

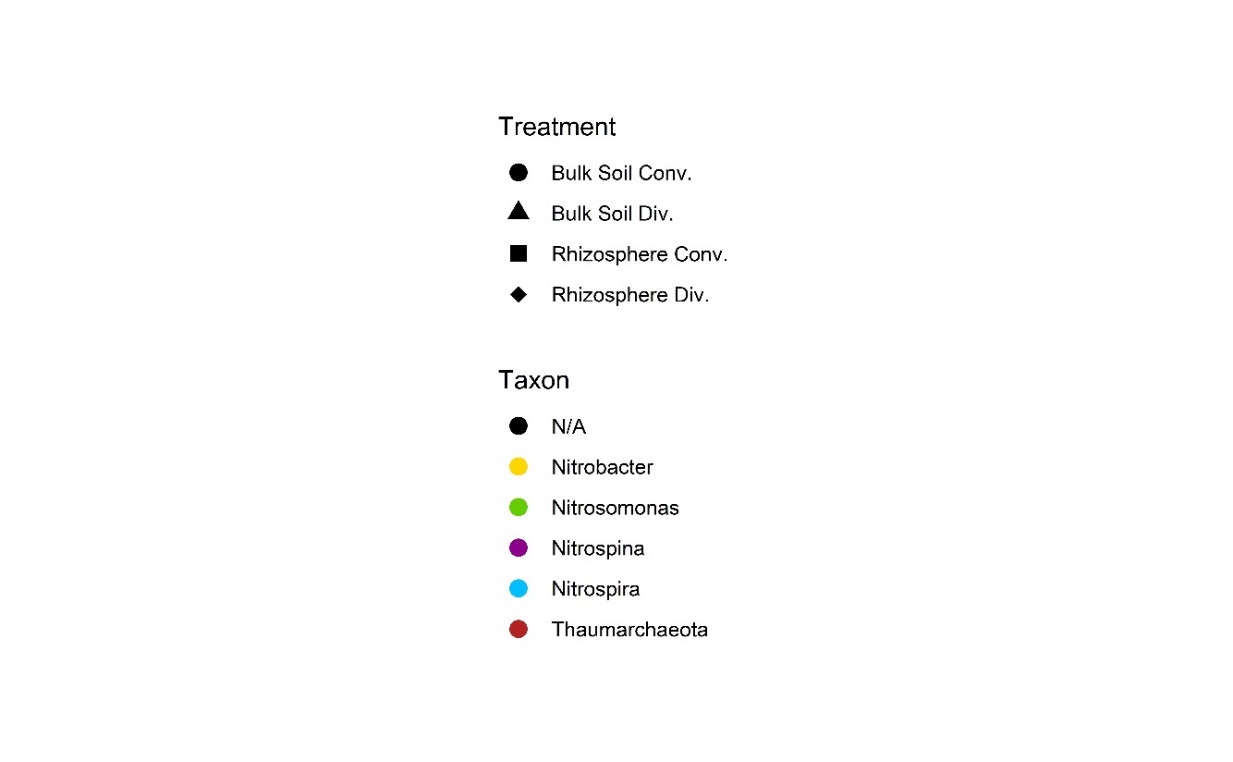


**Figure S1** Cumulative feature importance of random forest with inclusion of ammonia oxidizer *amoA* gene abundance. Features comprising approximately 90% variance are shown. Circles: Conventional bulk soil: Triangles: Diversified bulk soil; Squares: Conventional rhizosphere; Diamonds: Diversified rhizosphere. 1: AOA/AOB *amoA* abundance ratio; 2: AOA *amoA* abundance; 3: AOB *amoA* abundance; 4: NH_4_^+^ pool size


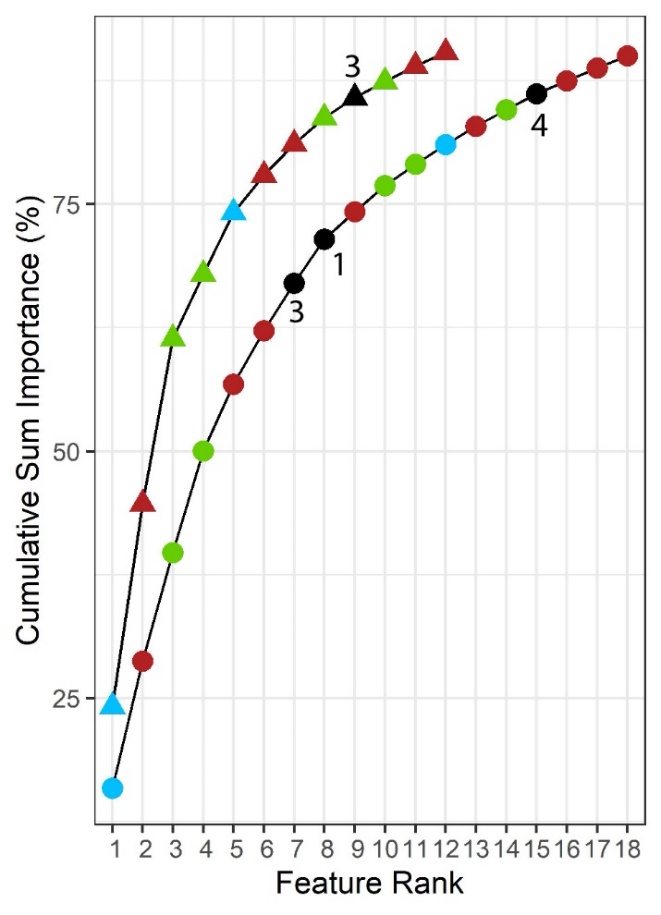

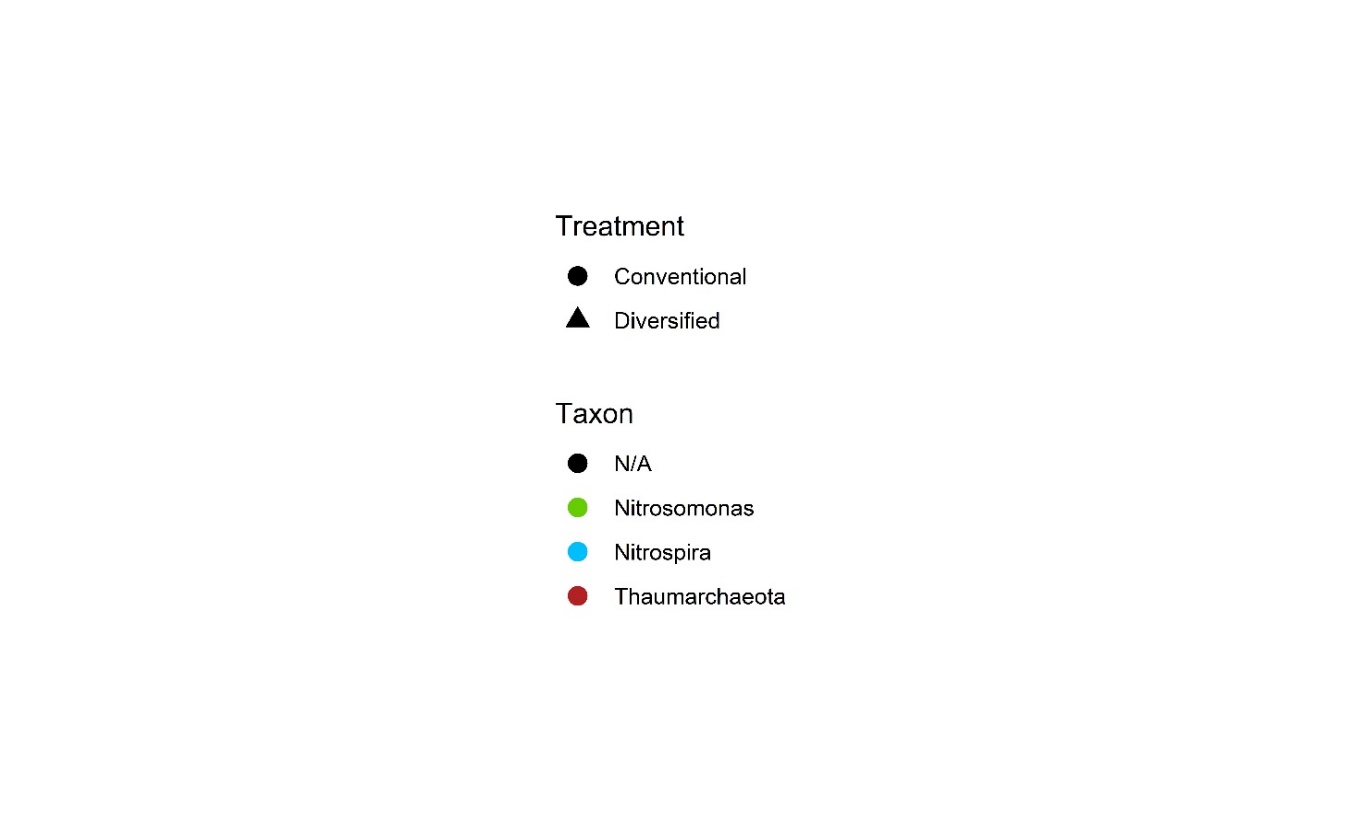


**Figure S2** Cumulative feature importance of random forest modeling of conventional and diversified cropping systems with inclusion of ammonia oxidizer *amoA* gene abundance. Features comprising approximately 90% variance are shown. Circles: Conventional: Triangles: Diversified. 1: AOA/AOB *amoA* abundance ratio; 2: AOA *amoA* abundance; 3: AOB *amoA* abundance; 4: NH_4_^+^ pool size

**
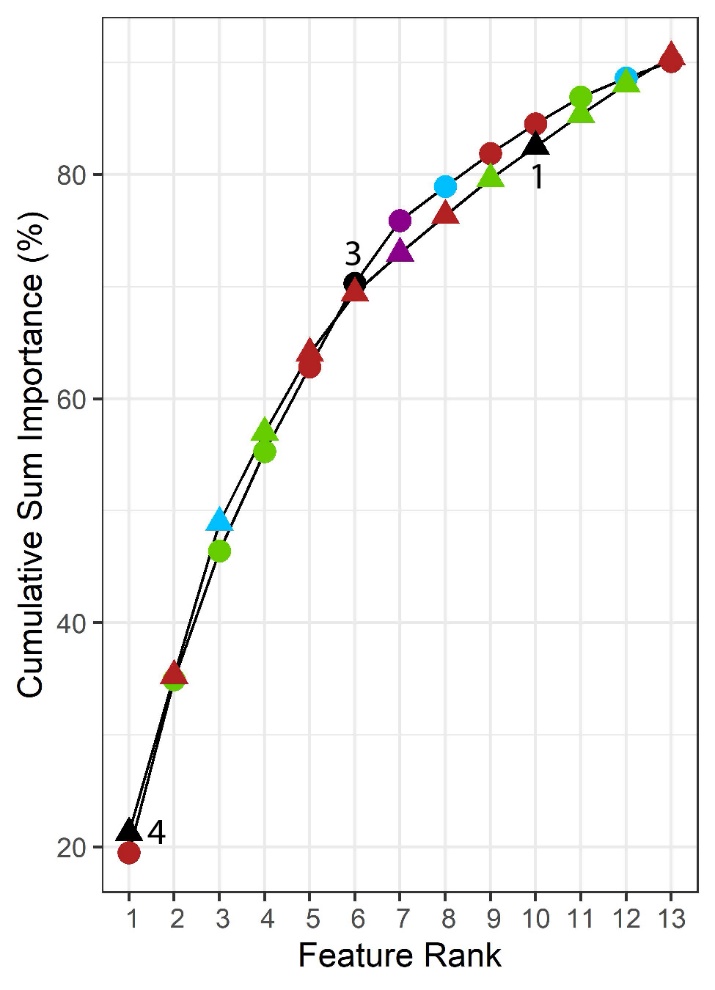
Figure S3** Cumulative feature importance of random forest modeling of bulk soil and rhizosphere with inclusion of ammonia oxidizer *amoA* gene abundance. Features comprising approximately 90% variance are shown. Circles: Bulk soil: Triangles: Rhizosphere. 1: AOA/AOB *amoA* abundance ratio; 2: AOA *amoA* abundance; 3: AOB *amoA* abundance; 4: NH_4_^+^ pool size
